# Supplementary material for: Cognitive-behavioral therapy for the improvement of negative symptoms and functioning in schizophrenia: A systematic review and meta-analysis of randomized controlled trials
Source: PLoS One. 2025 May 20;20(5):e0324685. doi: 10.1371/journal.pone.0324685 (PMC12091889; doi:10.1371/journal.pone.0324685)
Supplement: S1 — (DOCX) [file pone.0324685.s001.docx]

**S1 Search strategy**

| **Database** | **Search Strategy** | **Results** |
| --- | --- | --- |
|  | **Pubmed** |  |
|  |  |  |
| #1 | "Schizophrenia"[Mesh] | 117751 |
| #2 | ((((((Schizophrenias[Title/Abstract]) OR (Dementia Praecox[Title/Abstract])) OR (Schizophrenic Disorders[Title/Abstract])) OR (Disorder, Schizophrenic[Title/Abstract])) OR (Disorders, Schizophrenic[Title/Abstract])) OR (Schizophrenic Disorder[Title/Abstract])) OR (schizophrenic syndrome[Title/Abstract]) | 992,394 |
| #3 | #1 OR #2 | 118263 |
| #4 | "Cognitive Behavioral Therapy"[Mesh] | 38863 |
| #5 | (((((((((((((((((((((((((((((Behavioral Therapies, Cognitive[Title/Abstract]) OR (Behavioral Therapy, Cognitive[Title/Abstract])) OR (Cognitive Behavioral Therapies[Title/Abstract])) OR (Therapies, Cognitive Behavioral[Title/Abstract])) OR (Therapy, Cognitive Behavioral[Title/Abstract])) OR (Cognition Therapy[Title/Abstract])) OR (Cognition Therapies[Title/Abstract])) OR (Therapies, Cognition[Title/Abstract])) OR (Therapy, Cognitive Behavior[Title/Abstract])) OR (Behavior Therapies, Cognitive[Title/Abstract])) OR (Cognitive Behavior Therapies[Title/Abstract])) OR (Therapies, Cognitive Behavior[Title/Abstract])) OR (Therapy, Cognition[Title/Abstract])) OR (Behavior Therapy, Cognitive[Title/Abstract])) OR (Cognitive Behavior Therapy[Title/Abstract])) OR (Cognitive Psychotherapy[Title/Abstract])) OR (Cognitive Psychotherapies[Title/Abstract])) OR (Psychotherapies, Cognitive[Title/Abstract])) OR (Psychotherapy, Cognitive[Title/Abstract])) OR (Therapy, Cognitive[Title/Abstract])) OR (Cognitive Therapies[Title/Abstract])) OR (Cognitive Therapies[Title/Abstract])) OR (Therapies, Cognitive[Title/Abstract])) OR (Cognitive Behaviour Therapy[Title/Abstract])) OR (Behaviour Therapies, Cognitive[Title/Abstract])) OR (Behaviour Therapy, Cognitive[Title/Abstract])) OR (Cognitive Behaviour Therapies[Title/Abstract])) OR (Therapies, Cognitive Behaviour[Title/Abstract])) OR (Therapy, Cognitive Behaviour[Title/Abstract])) OR (Cognitive Therapy[Title/Abstract]) | 44430 |
| #6 | #4 OR #5 | 75995 |
| #7 | ((((((randomized controlled trial[Publication Type]) OR (randomized[Title/Abstract])) OR (placebo[Title/Abstract])) OR (drug therapy[Title/Abstract])) OR (randomly[Title/Abstract])) OR (trial[Title/Abstract])) OR (groups[Title/Abstract]) | 3985394 |
| #8 | #3 AND #6 AND #7 | 1461 |
|  | **Web of Science core collection** |  |
| #1 | TS=(Schizophrenia or Schizophrenias or Dementia Praecox or Schizophrenic Disorders or Disorder, Schizophrenic or Disorders, Schizophrenic or Schizophrenic Disorder or schizophrenic syndrome) | 219542 |
| #2 | TS=(Behavioral Therapies, Cognitive or Behavioral Therapy, Cognitive or Cognitive Behavioral Therapies or Therapies, Cognitive Behavioral or Therapy, Cognitive Behavioral or Cognition Therapy or Cognition Therapies or Therapies, Cognition or Therapy, Cognitive Behavior or Behavior Therapies, Cognitive or Cognitive Behavior Therapies or Therapies, Cognitive Behavior or Therapy, Cognition or Behavior Therapy, Cognitive or Cognitive Behavior Therapy or Cognitive Psychotherapy or Cognitive Psychotherapies or Psychotherapies, Cognitive or Psychotherapy, Cognitive or Therapy, Cognitive or Cognitive Therapies or Therapies, Cognitive or Cognitive Behaviour Therapy or Behaviour Therapies, Cognitive or Behaviour Therapy, Cognitive or Cognitive Behaviour Therapies or Therapies, Cognitive Behaviour or Therapy, Cognitive Behaviour or Cognitive Therapy) | 115344 |
| #3 | TS=(randomized controlled trial OR randomized OR placebo OR drug therapy OR randomly OR trial) | 3268900 |
| #4 | #1 AND #2 AND #3 | 3448 |
|  | **Embase** |  |
| #1 | 'schizophrenia'/exp | 267121 |
| #2 | 'schizophrenias':ab,ti OR 'dementia praecox':ab,ti OR 'schizophrenic disorders':ab,ti OR 'disorder, schizophrenic':ab,ti OR 'disorders, schizophrenic':ab,ti OR 'schizophrenic disorder':ab,ti OR 'schizophrenic syndrome':ab,ti | 3071 |
| #3 | #1 OR #2 | 267315 |
| #4 | 'behavioral therapies, cognitive':ab,ti OR 'behavioral therapy, cognitive':ab,ti OR 'cognitive behavioral therapies':ab,ti OR 'therapies, cognitive behavioral':ab,ti OR 'therapy, cognitive behavioral':ab,ti OR 'cognition therapy':ab,ti OR 'cognition therapies':ab,ti OR 'therapies, cognition':ab,ti OR 'therapy, cognitive behavior':ab,ti OR 'behavior therapies, cognitive':ab,ti OR 'cognitive behavior therapies':ab,ti OR 'therapies, cognitive behavior':ab,ti OR 'therapy, cognition':ab,ti OR 'behavior therapy, cognitive':ab,ti OR 'cognitive behavior therapy':ab,ti OR 'cognitive psychotherapy':ab,ti OR 'cognitive psychotherapies':ab,ti OR 'psychotherapies, cognitive':ab,ti OR 'psychotherapy, cognitive':ab,ti OR 'therapy, cognitive':ab,ti OR 'cognitive therapies':ab,ti OR 'therapies, cognitive':ab,ti OR 'cognitive behaviour therapy':ab,ti OR 'behaviour therapies, cognitive':ab,ti OR 'behaviour therapy, cognitive':ab,ti OR 'cognitive behaviour therapies':ab,ti OR 'therapies, cognitive behaviour':ab,ti OR 'therapy, cognitive behaviour':ab,ti OR 'cognitive therapy':ab,ti | 14028 |
| #5 | 'randomized controlled trial'/exp | 837972 |
| #6 | 'randomized controlled trial':ti,ab,kw OR randomized:ti,ab,kw OR placebo:ti,ab,kw OR 'drug therapy':ti,ab,kw OR randomly:ti,ab,kw OR trial:ti,ab,kw | 2353437 |
| #7 | #5 OR #6 | 2476431 |
| #8 | #3 AND #4 AND #7 | 371 |
|  | **CINAHL Complete** |  |
| S1 | (MH "Schizophrenia") | 29495 |
| S2 | Schizophrenias or Dementia Praecox or Schizophrenic Disorders or Disorder, Schizophrenic or Disorders, Schizophrenic or Schizophrenic Disorder or schizophrenic syndrome | 38347 |
| S3 | S1 OR S2 | 38347 |
| S4 | (MH "Cognitive Therapy") OR (MH "Cognitive Remediation") | 22023 |
| S5 | Behavioral Therapies, Cognitive or Behavioral Therapy, Cognitive or Cognitive Behavioral Therapies or Therapies, Cognitive Behavioral or Therapy, Cognitive Behavioral or Cognition Therapy or Cognition Therapies or Therapies, Cognition or Therapy, Cognitive Behavior or Behavior Therapies, Cognitive or Cognitive Behavior Therapies or Therapies, Cognitive Behavior or Therapy, Cognition or Behavior Therapy, Cognitive or Cognitive Behavior Therapy or Cognitive Psychotherapy or Cognitive Psychotherapies or Psychotherapies, Cognitive or Psychotherapy, Cognitive or Therapy, Cognitive or Cognitive Therapies or Therapies, Cognitive or Cognitive Behaviour Therapy or Behaviour Therapies, Cognitive or Behaviour Therapy, Cognitive or Cognitive Behaviour Therapies or Therapies, Cognitive Behaviour or Therapy, Cognitive Behaviour or Cognitive Therapy | 33002 |
| S6 | S4 OR S5 | 33115 |
| S7 | (MH "Randomized Controlled Trials") | 144056 |
| S8 | randomized controlled trial OR randomized O placebo OR drug therapy OR randomly OR trial | 868957 |
| S9 | S7 OR S8 | 868957 |
| S10 | S3 AND S6 AND S9 | 559 |
|  | **APA PsycInfo (EBSCO)** |  |
| S1 | DE "Schizophrenia" | 109401 |
| S2 | Schizophrenias or Dementia Praecox or Schizophrenic Disorders or Disorder, Schizophrenic or Disorders, Schizophrenic or Schizophrenic Disorder or schizophrenic syndrome | 169917 |
| S3 | S1 OR S2 | 169917 |
| S4 | (DE "Cognitive Therapy") OR (DE "Cognitive Remediation") | 25244 |
| S5 | Behavioral Therapies, Cognitive or Behavioral Therapy, Cognitive or Cognitive Behavioral Therapies or Therapies, Cognitive Behavioral or Therapy, Cognitive Behavioral or Cognition Therapy or Cognition Therapies or Therapies, Cognition or Therapy, Cognitive Behavior or Behavior Therapies, Cognitive or Cognitive Behavior Therapies or Therapies, Cognitive Behavior or Therapy, Cognition or Behavior Therapy, Cognitive or Cognitive Behavior Therapy or Cognitive Psychotherapy or Cognitive Psychotherapies or Psychotherapies, Cognitive or Psychotherapy, Cognitive or Therapy, Cognitive or Cognitive Therapies or Therapies, Cognitive or Cognitive Behaviour Therapy or Behaviour Therapies, Cognitive or Behaviour Therapy, Cognitive or Cognitive Behaviour Therapies or Therapies, Cognitive Behaviour or Therapy, Cognitive Behaviour or Cognitive Therapy | 134258 |
| S6 | S4 OR S5 | 134622 |
| S7 | DE "Randomized Controlled Trials" | 1096 |
| S8 | randomized controlled trial OR randomized O placebo OR drug therapy OR randomly OR trial | 455138 |
| S9 | S7 OR S8 | 455138 |
| S10 | S3 AND S6 AND S9 | 5266 |
|  | **MEDLINE (EBSCO)** |  |
| S1 | (MH "Schizophrenia") | 113950 |
| S2 | Schizophrenias or Dementia Praecox or Schizophrenic Disorders or Disorder, Schizophrenic or Disorders, Schizophrenic or Schizophrenic Disorder or schizophrenic syndrome | 170804 |
| S3 | S1 OR S2 | 170804 |
| S4 | (MH "Cognitive Behavioral Therapy") OR (MH "Cognitive Remediation") | 31927 |
| S5 | Behavioral Therapies, Cognitive or Behavioral Therapy, Cognitive or Cognitive Behavioral Therapies or Therapies, Cognitive Behavioral or Therapy, Cognitive Behavioral or Cognition Therapy or Cognition Therapies or Therapies, Cognition or Therapy, Cognitive Behavior or Behavior Therapies, Cognitive or Cognitive Behavior Therapies or Therapies, Cognitive Behavior or Therapy, Cognition or Behavior Therapy, Cognitive or Cognitive Behavior Therapy or Cognitive Psychotherapy or Cognitive Psychotherapies or Psychotherapies, Cognitive or Psychotherapy, Cognitive or Therapy, Cognitive or Cognitive Therapies or Therapies, Cognitive or Cognitive Behaviour Therapy or Behaviour Therapies, Cognitive or Behaviour Therapy, Cognitive or Cognitive Behaviour Therapies or Therapies, Cognitive Behaviour or Therapy, Cognitive Behaviour or Cognitive Therapy | 55575 |
| S6 | S4 OR S5 | 56001 |
| S7 | (MH "Randomized Controlled Trials as Topic") | 171249 |
| S8 | randomized controlled trial OR randomized O placebo OR drug therapy OR randomly OR trial | 4251133 |
| S9 | S7 OR S8 | 4251133 |
| S10 | S3 AND S6 AND S9 | 1357 |
|  | **Cochrane Library** |  |
| #1 | (Schizophrenia):ti,ab,kw or (Schizophrenias):ti,ab,kw or (Dementia Praecox):ti,ab,kw or (Schizophrenic Disorders):ti,ab,kw or (Disorder, Schizophrenic):ti,ab,kw or (Disorders, Schizophrenic):ti,ab,kw or (Schizophrenic Disorder):ti,ab,kw or (schizophrenic syndrome):ti,ab,kw | 10509 |
| #2 | (Behavioral Therapies, Cognitive):ti,ab,kw or (Behavioral Therapy, Cognitive):ti,ab,kw or (Cognitive Behavioral Therapies):ti,ab,kw or (Therapies, Cognitive Behavioral):ti,ab,kw or (Therapy, Cognitive Behavioral):ti,ab,kw or (Cognition Therapy):ti,ab,kw or (Cognition Therapies):ti,ab,kw or (Therapies, Cognition):ti,ab,kw or (Therapy, Cognitive Behavior):ti,ab,kw or (Behavior Therapies, Cognitive):ti,ab,kw or (Cognitive Behavior Therapies):ti,ab,kw or (Therapies, Cognitive Behavior):ti,ab,kw or (Therapy, Cognition):ti,ab,kw or (Behavior Therapy, Cognitive):ti,ab,kw or (Cognitive Behavior Therapy):ti,ab,kw or (Cognitive Psychotherapy):ti,ab,kw or (Cognitive Psychotherapies):ti,ab,kw or (Psychotherapies, Cognitive):ti,ab,kw or (Psychotherapy, Cognitive):ti,ab,kw or (Therapy, Cognitive):ti,ab,kw or (Cognitive Therapies):ti,ab,kw or (Therapies, Cognitive):ti,ab,kw or (Cognitive Behaviour Therapy):ti,ab,kw or (Behaviour Therapies, Cognitive):ti,ab,kw or (Behaviour Therapy, Cognitive):ti,ab,kw or (Cognitive Behaviour Therapies):ti,ab,kw or (Therapies, Cognitive Behaviour):ti,ab,kw or (Therapy, Cognitive Behaviour):ti,ab,kw or (Cognitive Therapy):ti,ab,kw | 57975 |
| #3 | (randomized controlled trial):ti,ab,kw OR (randomized):ti,ab,kw OR (placebo):ti,ab,kw OR (drug therapy):ti,ab,kw OR (randomly):ti,ab,kw OR (trial):ti,ab,kw | 1602863 |
| #4 | #3 and #4 and #5 | 1844 |
|  | CNKI |  |
| #1 | （主题：精神分裂症）OR（主题：阴性症状）OR（主题：精神分裂症阴性症状） | 54471 |
| #2 | （主题：认知行为疗法）OR（主题：认知行为） | 19956 |
| #3 | （主题：随机对照试验）OR（主题：随机） | 429472 |
| #4 | #1 AND #2 AND #3 | 13 |
|  | WanFang |  |
| #1 | 主题:(精神分裂症) or 主题:(阴性症状) or 主题:(精神分裂症阴性症状) | 67785 |
| #2 | 主题:("认知行为疗法") or 主题:("认知行为") | 12965 |
| #3 | 主题:("随机对照试验") or 主题:("随机") | 2918076 |
| #4 | #1 AND #2 AND #3 | 125 |
|  | SinoMed |  |
| #1 | "精神分裂症"[常用字段:智能] OR "阴性症状"[常用字段:智能] OR "精神分裂症阴性症状"[常用字段:智能] | 47800 |
| #2 | "认知行为疗法"[常用字段:智能] OR "认知行为"[常用字段:智能] | 13292 |
| #3 | "随机对照试验"[常用字段:智能] OR "随机"[常用字段:智能] | 2038767 |
| #4 | #1 AND #2 AND #3 | 60 |
