# Supplementary material for: Cognitive-behavioral therapy for the improvement of negative symptoms and functioning in schizophrenia: A systematic review and meta-analysis of randomized controlled trials
Source: PLoS One. 2025 May 20;20(5):e0324685. doi: 10.1371/journal.pone.0324685 (PMC12091889; doi:10.1371/journal.pone.0324685)
Supplement: S2 — (DOCX) [file pone.0324685.s002.docx]

**S2 Table GRADE Assessment**

| Outcomes | Study  Design | Risk of  Bias | Inconsistency | Indirectness | Imprecision | Other  Considerations | No. of Participants | | Effect(95%CI) | Quality |
| --- | --- | --- | --- | --- | --- | --- | --- | --- | --- | --- |
|  |  |  |  |  |  |  | cognitive therapy | Control |  |  |
| PANSS-Negative Symptom | RCTs | Serious | No | No | No | No | 661 | 650 | MD=-1.65( -2.10 to-1.21) | Moderate |
| overall function | RCTs | Serious | No | No | No | Serious | 126 | 126 | MD=0.38(0.13 to 0.63) | Low |
| Social Skills | RCTs | Serious | No | No | No | Serious | 102 | 102 | SMD=0.87(0.58 to 1.16) | Low |
| Social Functioning | RCTs | Serious | No | No | No | Serious | 280 | 282 | SMD=0.19(0.03 to 0.36) | Low |

**cognitive therapy versus TAU**
