# Supplementary figures and images for: Cognitive-behavioral therapy for the improvement of negative symptoms and functioning in schizophrenia: A systematic review and meta-analysis of randomized controlled trials
Source: PLoS One. 2025 May 20;20(5):e0324685. doi: 10.1371/journal.pone.0324685 (PMC12091889; doi:10.1371/journal.pone.0324685)

### Funnel plot with pseudo 95% confidence limits

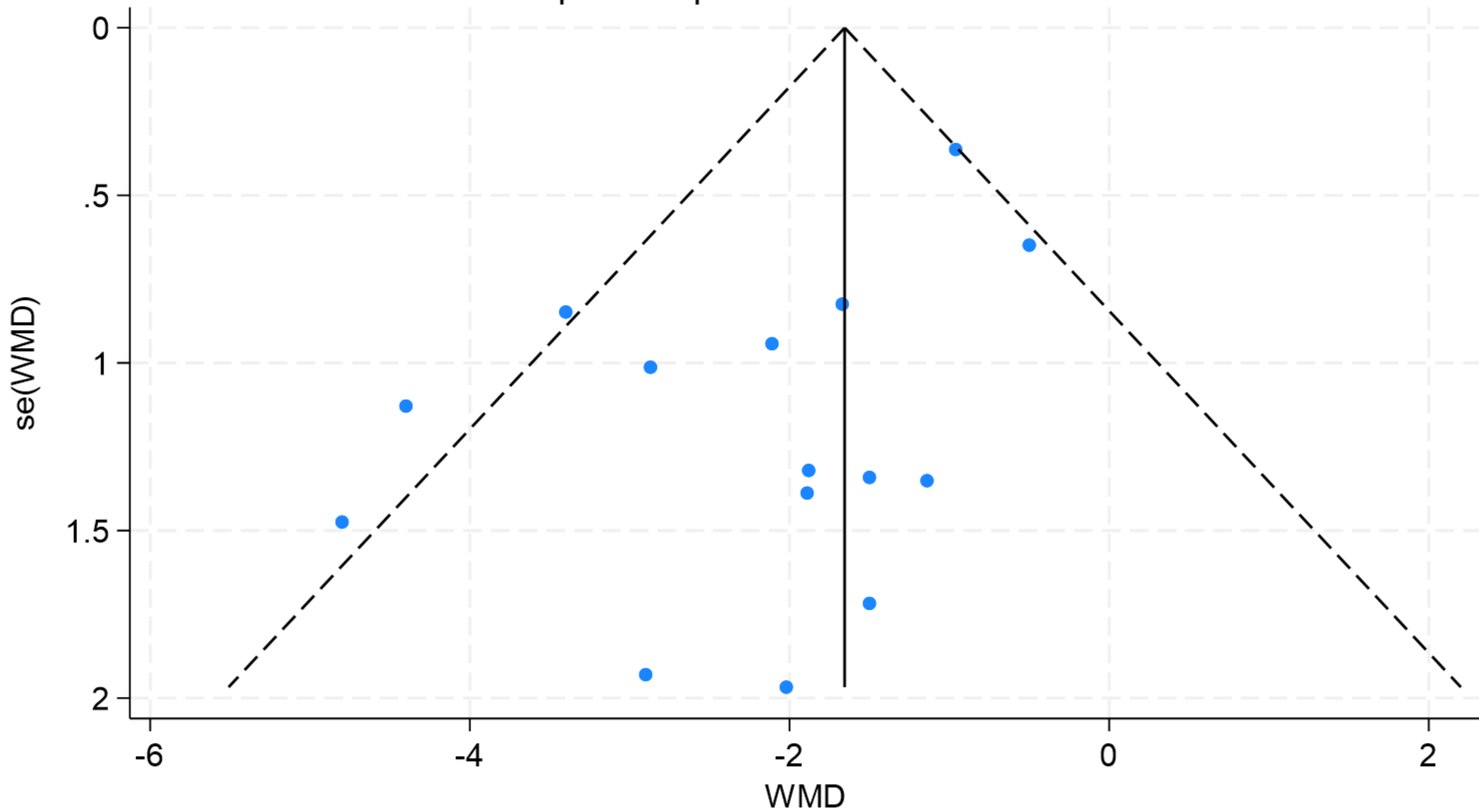

Supplement: S3 — (PDF) [file pone.0324685.s003.pdf]
