# Supplementary material for: Cognitive-behavioral therapy for the improvement of negative symptoms and functioning in schizophrenia: A systematic review and meta-analysis of randomized controlled trials
Source: PLoS One. 2025 May 20;20(5):e0324685. doi: 10.1371/journal.pone.0324685 (PMC12091889; doi:10.1371/journal.pone.0324685)
Supplement: S8 — (DOCX) [file pone.0324685.s008.docx]

| **Study** | **Random sequence generation (selection bias)** | **Allocation concealment (selection bias)** | **Blinding of participants and personnel (performance bias)** | **Blinding of outcome assessment (detection bias)** | **Incomplete outcome data (attrition bias)** | **Selective reporting (reporting bias)** | **Other bias** |
| --- | --- | --- | --- | --- | --- | --- | --- |
| Anthony (2)  et al,  2018 | Low risk | Low risk | Low risk | Low risk | Low risk | Low risk | Low risk |
| Anthony et al,  2018 | Low risk | Low risk | Low risk | Low risk | Low risk | Low risk | Low risk |
| Barrowclough  et al,  2001 | Low risk | Unclear risk | Low risk | Low risk | Low risk | Low risk | Low risk |
| Barrowclough  et al,  2006 | Low risk | Unclear risk | High risk | High risk | Low risk | Low risk | Low risk |
| Dandan Chen  et al,  2024 | Low risk | Unclear risk | High risk | High risk | Low risk | Low risk | Low risk |
| Faxia Peng et al,  2023 | Low risk | Unclear risk | High risk | High risk | Low risk | Low risk | Low risk |
| Gumley et al,  2003 | Low risk | Unclear risk | High risk | High risk | Low risk | Low risk | Low risk |
| Yan Jun et al,  2024 | Low risk | Unclear risk | High risk | High risk | Low risk | Low risk | Low risk |
| Morrison et al,  2014 | Low risk | Low risk | Low risk | Low risk | Low risk | Low risk | Low risk |
| Müller et al, 2020 | Low risk | Unclear risk | Low risk | Low risk | Low risk | Low risk | Low risk |
| Penn et al,  2021 | Low risk | Unclear risk | High risk | High risk | Low risk | Low risk | Low risk |
| Peters et al, 2010 | Low risk | Unclear risk | Low risk | Low risk | Low risk | Low risk | Low risk |
| Rector et al, 2014 | Low risk | Unclear risk | Low risk | Low risk | Low risk | Low risk | Low risk |
| Sönmez et al,  2020 | Low risk | Unclear risk | Low risk | Low risk | Low risk | Low risk | Low risk |
| Tarrier et al,  2004 | Low risk | Low risk | Low risk | Low risk | Low risk | Low risk | Low risk |

**S7 Table. Methodological quality of the trials**
