# Supplementary material for: Cognitive-behavioral therapy for the improvement of negative symptoms and functioning in schizophrenia: A systematic review and meta-analysis of randomized controlled trials
Source: PLoS One. 2025 May 20;20(5):e0324685. doi: 10.1371/journal.pone.0324685 (PMC12091889; doi:10.1371/journal.pone.0324685)
Supplement: Attachment — Submitted filename: Reviewer Report_20Feb2025.pdf [file pone.0324685.s009.pdf]

Feb 20, 2025

Peer review report

Dear Editor,

Thank you for the opportunity to review this manuscript titled "*Cognitive Therapy for the Improvement of Negative Symptoms in Schizophrenia: A Systematic Review and Meta-Analysis of Randomized Controlled Trials.*" I have carefully read through the document and provide my detailed comments below.

This is a systematic review and meta-analysis of randomized controlled trials investigating the effect of "cognitive therapies" on the symptoms of schizophrenia. The authors report a significant reduction in symptoms across multiple domains when compared to treatment as usual. Demonstrating the therapeutic effect of psychosocial interventions in schizophrenia is important, not only because they are effective in the treatment of various symptom domains, but also because they are not associated with the side effect burden of antipsychotic medications. he evidence is limited, as the authors put it, by the lack of targeted interventions and the absence of long-term studies, which are strongly needed.

I believe the current manuscript must undergo the following major revisions in order to be accepted for publication.

Major Points:

1. Although the title specifies "negative symptoms" as the outcome under investigation, the results also include positive symptoms, depressive symptoms, and functioning. I suggest that the authors address this issue by maintaining their focus solely on negative symptoms and functioning. This is because the effect of the interventions under investigation on positive symptoms has been reported elsewhere and is not the focus of this project, unless the authors intend to explore the relationship between reductions in positive symptoms and negative symptoms. In that case, the authors should demonstrate that the reduction in negative symptoms is strongly correlated with positive symptom reduction. This point is related to subsequent comments (No. 4 and 5).

2. A recently published systematic review and meta-analysis has investigated the response to cognitive behavioral therapy (CBT) for negative symptoms in patients with schizophrenia:
    - Xu F, Xu S. Cognitive-behavioral therapy for negative symptoms of schizophrenia: A systematic review and meta-analysis. Medicine (Baltimore). 2024;103(36):e39572. doi:10.1097/MD.0000000000039572

That study restricted its search to CBT and included nonrandomized trials, whereas this systematic review included both CBT and cognitive remediation therapy (CRT). CRT has been investigated in an older systematic review and meta-analysis of randomized trials:

- Cella M, Preti A, Edwards C, Dow T, Wykes T. Cognitive remediation for negative symptoms of schizophrenia: A network meta-analysis. Clin Psychol Rev. 2017;52:43-51. doi:10.1016/j.cpr.2016.11.009

Both of these systematic reviews concluded that CBT and CRT are effective in reducing the burden of negative symptoms, notwithstanding the limitations of the included studies. However, the present systematic review grouped CBT and CRT together under one category of intervention and analyzed them jointly. I suggest dedicating a portion of the introduction to justifying this methodology. Specifically, why should a systematic review combine both CBT and CRT interventions when the techniques and primary focuses of these therapies are markedly different? Sharing the label "cognitive" does not warrant this combination. As the authors know, CBT targets automatic thoughts and biases that influence emotions and behavior. It involves the use of thought records and behavioral activation, aiming to reduce distress and disability associated with psychotic symptoms. CRT, on the other hand, is a training-based intervention that aims to improve cognitive processes such as attention, memory, executive function, social cognition, or metacognition to overcome cognitive deficits.

In addition to the previous point (No. 1), I believe this issue is a major concern in the current manuscript. The authors might want to either restrict their investigation solely to CRT (since a recently published meta-analysis already covers CBT) or provide a convincing rationale for grouping both CBT and CRT in a single meta-analysis.

Minor Points:

2. In the introduction, the authors state that negative symptoms encompass "psychomotor retardation and attention deficits." This is not accurate. The latest conceptualization of the negative domain of schizophrenia encompasses five domains to better distinguish it from psychomotor symptoms and, especially, cognitive domains. Please refer to:
    - Kirkpatrick B, Fenton WS, Carpenter WT Jr, Marder SR. The NIMH-MATRICS consensus statement on negative symptoms. Schizophr Bull. 2006;32(2):214-219. doi:10.1093/schbul/sbj053
    - Strauss GP, Ahmed AO, Young JW, Kirkpatrick B. Reconsidering the Latent Structure of Negative Symptoms in Schizophrenia: A Review of Evidence Supporting the 5 Consensus Domains. Schizophr Bull. 2019;45(4):725-729. doi:10.1093/schbul/sby169

3. In the introduction, the authors explain that antipsychotic treatment is not "without potential side effects." However, this is not why they have limited use in targeting negative symptoms. The actual reason is that antipsychotic medications have very limited efficacy in reducing negative symptom severity and lack established evidence for treating primary and enduring negative symptoms. This is precisely the problem facing therapeutic research for negative symptoms, that is, the lack of evidence for available interventions. Kindly refer to:
    - Fusar-Poli P, Papanastasiou E, Stahl D, et al. Treatments of Negative Symptoms in Schizophrenia: Meta-Analysis of 168 Randomized Placebo-Controlled Trials [published correction appears in Schizophr Bull. 2022 May 7;48(3):721. doi: 10.1093/schbul/sbz071.]. Schizophr Bull. 2015;41(4):892-899. doi:10.1093/schbul/sbu170
    - Krause M, Zhu Y, Huhn M, et al. Antipsychotic drugs for patients with schizophrenia and predominant or prominent negative symptoms: a systematic review and meta-analysis. Eur Arch Psychiatry Clin Neurosci. 2018;268(7):625-639. doi:10.1007/s00406-018-0869-3
    - Remington G, Foussias G, Fervaha G, et al. Treating Negative Symptoms in Schizophrenia: an Update. Curr Treat Options Psychiatry. 2016;3:133-150. doi:10.1007/s40501-016-0075-8

4. In the results, the authors state that *"The subgroup differences (P = 0.68)."* I am not sure what this means. The subgroup analysis indicates that the reduction in negative symptoms is not consistently significant across time. In the abstract and discussion, the authors argue that this lack of effect across time is *"due to the lack of cognitive therapy specifically targeting negative symptoms."* This is merely a conjecture. The results, in my view, suggest that CBT and CRT do not effectively improve primary and enduring negative symptoms, which are the main targets of negative symptom treatment in schizophrenia. This needs to be mentioned as a possible interpretation of the results. After all, secondary negative symptoms in schizophrenia also respond to antipsychotic medications. Please see:
    - Correll CU, Schooler NR. Negative Symptoms in Schizophrenia: A Review and Clinical Guide for Recognition, Assessment, and Treatment. Neuropsychiatr Dis Treat. 2020;16:519-534. Published 2020 Feb 21. doi:10.2147/NDT.S225643

5. Also related to the previous point is the finding in this systematic review that total PANSS scores tend to decrease with CBT or CRT, which further raises the possibility that improvement in negative symptoms is linked to improvement in other domains—that is, the effect may primarily be on secondary negative symptoms. This issue needs to be discussed in detail. Unfortunately, due to this problem, the results do not advance the field of negative symptom therapeutics, except for the finding that there is no significant difference across short- and long-term comparisons, which, in my view, indicates a lack of benefit.

Thank you once again for the opportunity to review this manuscript.
