# Supplementary material for: Cognitive-behavioral therapy for the improvement of negative symptoms and functioning in schizophrenia: A systematic review and meta-analysis of randomized controlled trials
Source: PLoS One. 2025 May 20;20(5):e0324685. doi: 10.1371/journal.pone.0324685 (PMC12091889; doi:10.1371/journal.pone.0324685)
Supplement: Attachment — Submitted filename: Response to Reviews.docx [file pone.0324685.s011.docx]

Dear Editors and Reviewers:

Thank you for your letter and for the reviewers' comments concerning our manuscript entitled “Cognitive Therapy for the Improvement of Negative Symptoms in Schizophrenia: A Systematic Review and Meta-Analysis of Randomized Controlled Trials” (Manuscript ID: PONE-D-24-37272). Those comments are all valuable and very helpful for revising and improving our paper, as well as the important guiding significance to our research. We have studied comments carefully and have made correction which we hope to meet with approval. Revised portion are marked in red (with track and highlighted changes) in the paper. The main corrections in the paper and the responses to the reviewer’s comments are as follows. Please don’t hesitate to contact us in case there are any problems regarding this manuscript.

**Reviewer #1**

**To begin with, we thank the reviewer for the effort and time put into the review of the manuscript, special thanks to you for your good comments and warm work earnestly.**

In terms of content the following;

**Comment 1:**

I will suggest changing the language in conclusion section of abstract where the line “no significant differences were observed in short term,….” Appears to indicate there was no improved from cognitive treatment and should be changed to clarify there were no differences among the 3 subsets of treatment with respect to improvement of negative symptoms.

**Reply 1:**

Thank you for your valuable feedback. We appreciate your suggestion to clarify the language in the conclusion section of the abstract. Based on your comment, we have revised the sentence to more accurately reflect that there were no significant differences among the three subsets of treatment (short-term, medium-term, and long-term) with respect to the improvement of negative symptoms, rather than implying a lack of improvement from cognitive therapy.

The revised text now reads:

"The results indicate that cognitive behavioral therapy has a significant effect on improving the negative symptoms of schizophrenia and is markedly superior to Treatment as Usual (TAU). Moreover, all three sub-treatment approaches (short-term, medium-term, and long-term) can sustainably and significantly improve negative symptoms of schizophrenia. Future research should focus on developing and evaluating cognitive therapies targeting negative symptoms, providing more reliable evidence and applying these research findings to clinical practice."

We believe this revision addresses your concern and improves the clarity of our findings. Thank you again for your thoughtful review.

**Changes in the text: (Abstract: see marked manuscript Page 4, line 77-83)**

**Comment 2:**

I think it would be helpful to have a conclusion and discussion section about the future, limitations as well as what else would you have liked to know at the end of the paper.

**Reply 2:** Thank you very much for your valuable suggestions. We have added discussions on future research directions, limitations of the study, and areas for further exploration in the discussion section. These additions aim to provide readers with a more comprehensive perspective and guide future research. We greatly appreciate your contribution to enhancing the quality of our paper!

**Changes in the text:** **(Discussion: see marked manuscript Page 14-16 line 381-494)**

**Comment 3:** Otherwise, it is well designed study.

**Reply 3:** Thank you very much for your valuable comments. We truly appreciate your recognition that “it is well designed study.” Your feedback is highly important to us, and we will continue to refine our work based on your suggestions.

Thank you again for taking the time to review our manuscript. We look forward to further opportunities for discussion and improvement.

**Reviewer #2**

**To begin with, we thank the reviewer for the effort and time put into the review of the manuscript, special thanks to you for your good comments and warm work earnestly.**

**General comments:**

This is a systematic review and meta-analysis of randomized controlled trials investigating the effect of “cognitive therapies” on the symptoms of schizophrenia. The authors report a significant reduction in symptoms across multiple domains when compared to treatment as usual. Demonstrating the therapeutic effect of psychosocial interventions in schizophrenia is important, not only because they are effective in the treatment of various symptom domains, but also because they are not associated with the side effect burden of antipsychotic medications. he evidence is limited, as the authors put it, by the lack of targeted interventions and the absence of long-term studies, which are strongly needed.

**Reply:**

Thank you for your thoughtful and constructive feedback on our systematic review and meta-analysis. We greatly appreciate your recognition of the importance of demonstrating the therapeutic effects of psychosocial interventions, such as cognitive therapies, in the treatment of schizophrenia. We agree that these interventions not only show efficacy across multiple symptom domains but also offer a valuable alternative to antipsychotic medications by avoiding their associated side effect burden.

We also acknowledge your observation regarding the limitations of the current evidence, particularly the lack of targeted interventions and the absence of long-term studies. These points are indeed critical, and we have highlighted them in the discussion section of our manuscript. We fully agree that future research should prioritize the development of targeted cognitive therapies for specific symptom domains, as well as conduct long-term studies to better understand the sustained effects of these interventions.

Thank you again for your insightful comments, which have helped us strengthen the interpretation of our findings and underscore the need for further research in this area.

**Comment 1:**

Although the title specifies “negative symptoms” as the outcome under investigation, the results also include positive symptoms, depressive symptoms, and functioning. I suggest that the authors address this issue by maintaining their focus solely on negative symptoms and functioning. This is because the effect of the interventions under investigation on positive symptoms has been reported elsewhere and is not the focus of this project, unless the authors intend to explore the relationship between reductions in positive symptoms and negative symptoms. In that case, the authors should demonstrate that the reduction in negative symptoms is strongly correlated with positive symptom reduction. This point is related to subsequent comments (No. 4 and 5).

**Reply1:**

Thank you for your careful review and valuable feedback on our manuscript. We fully understand your suggestions regarding the title and the focus of our research. Indeed, while our study initially aimed to investigate the effects of cognitive therapy on negative symptoms, we also included data on positive symptoms, depressive symptoms, and functional improvement during the analysis. We agree with your perspective that the impact on positive symptoms has been extensively reported in other studies and is not the primary focus of this project.

Following your advice, we will adjust the focus of the paper to concentrate on the outcomes related to negative symptoms of schizophrenia, ensuring better alignment with the title and research objectives. We greatly appreciate this important comment, which will help us better focus on the research theme and enhance the clarity of the manuscript. If you have any further suggestions regarding the revised content, we would be more than happy to make additional improvements.

**Changes in the text:** **(Result: see marked manuscript Page 9-11, line 248-356)**

**Comment 2:**

A recently published systematic review and meta-analysis has investigated the response to cognitive behavioral therapy (CBT) for negative symptoms in patients with schizophrenia:
o Xu F, Xu S. Cognitive-behavioral therapy for negative symptoms of schizophrenia: A systematic review and meta-analysis. Medicine (Baltimore). 2024;103(36):e39572. doi:10.1097/MD.0000000000039572
That study restricted its search to CBT and included nonrandomized trials, whereas this systematic review included both CBT and cognitive remediation therapy (CRT). CRT has been investigated in an older systematic review and meta-analysis of randomized trials:
o Cella M, Preti A, Edwards C, Dow T, Wykes T. Cognitive remediation for negative symptoms of schizophrenia: A network meta-analysis. Clin Psychol Rev. 2017;52:43-51. doi:10.1016/j.cpr.2016.11.009

Both of these systematic reviews concluded that CBT and CRT are effective in reducing the burden of negative symptoms, notwithstanding the limitations of the included studies. However, the present systematic review grouped CBT and CRT together under one category of intervention and analyzed them jointly. I suggest dedicating a portion of the introduction to justifying this methodology. Specifically, why should a systematic review combine both CBT and CRT interventions when the techniques and primary focuses of these therapies are markedly different? Sharing the label “cognitive” does not warrant this combination. As the authors know, CBT targets automatic thoughts and biases that influence emotions and behavior. It involves the use of thought records and behavioral activation, aiming to reduce distress and disability associated with psychotic symptoms. CRT, on the other hand, is a training-based intervention that aims to improve cognitive processes such as attention, memory, executive function, social cognition, or metacognition to overcome cognitive deficits.
In addition to the previous point (No. 1), I believe this issue is a major concern in the current manuscript. The authors might want to either restrict their investigation solely to CRT (since a recently published meta-analysis already covers CBT) or provide a convincing rationale for grouping both CBT and CRT in a single meta-analysis.

**Reply2:**

Thank you for your valuable comments and suggestions. In response to your concerns regarding our combination of Cognitive Behavioral Therapy (CBT) and Cognitive Remediation Therapy (CRT) in the analysis, we have carefully reconsidered our approach. We acknowledge that, although both therapies share the label “cognitive,” they have distinct techniques and primary focuses, as you correctly pointed out. CBT targets automatic thoughts and biases, using thought records and behavioral activation to reduce distress and disability associated with psychotic symptoms. In contrast, CRT is a training-based intervention aimed at improving cognitive processes such as attention, memory, executive function, social cognition, or metacognition to address cognitive deficits. Given these differences, we agree that combining these interventions in a single meta-analysis may not be appropriate. Therefore, we have revised our systematic review to focus exclusively on CBT for the treatment of negative symptoms in schizophrenia. To ensure the accuracy and relevance of our findings, we have re-conducted the database search, limiting the intervention to CBT only. Specifically, we have excluded Cognitive Remediation Therapy (CRT) and restricted our investigation to Cognitive Behavioral Therapy (CBT) alone. Additionally, we have limited our analysis to Randomized Controlled Trials (RCTs) and included only studies with a control group receiving TAU (Treatment as Usual). The revised manuscript now specifically examines the efficacy of CBT for negative symptoms in schizophrenia, providing a clearer evaluation of its impact compared to TAU.

Thank you again for your valuable feedback. We believe that these changes have enhanced the rigor and clarity of our study.

**Changes in the text: (Title:** **Cognitive-behavioral therapy for the improvement of negative symptoms and functioning in schizophrenia: a systematic review and meta-analysis of randomized controlled trials. Result: Result: see marked manuscript Page 9-11, line 248-356.)**

**Comment 3:**

In the introduction, the authors state that negative symptoms encompass “psychomotor retardation and attention deficits.” This is not accurate. The latest conceptualization of the negative domain of schizophrenia encompasses five domains to better distinguish it from psychomotor symptoms and, especially, cognitive domains. Please refer to:
o Kirkpatrick B, Fenton WS, Carpenter WT Jr, Marder SR. The NIMH-MATRICS consensus statement on negative symptoms. Schizophr Bull. 2006;32(2):214-219. doi:10.1093/schbul/sbj053
o Strauss GP, Ahmed AO, Young JW, Kirkpatrick B. Reconsidering the Latent Structure of Negative Symptoms in Schizophrenia: A Review of Evidence Supporting the 5 Consensus Domains. Schizophr Bull. 2019;45(4):725-729. doi:10.1093/schbul/sby169

**Reply3**

We thank you for your careful review and valuable comments on our manuscript and the relevant literature provided. We fully agree with your comments and have made the corresponding revisions in the revised version.In the introduction section, we initially defined "negative symptoms" as "psychomotor retardation and attention deficits," which is indeed inaccurate. Based on the studies by Kirkpatrick et al. (2006) and Strauss et al. (2019), negative symptoms should encompass five core domains: blunted affect, alogia, avolition, asociality, and anhedonia. These domains aim to better distinguish negative symptoms from psychomotor symptoms and cognitive impairments.

In the revised manuscript, we have updated the introduction to clearly outline the five core domains of negative symptoms and have cited the aforementioned studies to support this perspective. We believe this revision will enhance the accuracy and scientific rigor of our work.

Once again, we sincerely appreciate your valuable input and look forward to your further feedback.

**Changes in the text:** They include blunted affect, alogia, avolition, asociality, and anhedonia, which can significantly impair patients' quality of life and hinder the rehabilitation process. **(Introduction: see marked manuscript Page 5, line 95-97)**

**Comment 4:**

In the introduction, the authors explain that antipsychotic treatment is not “without potential side effects.” However, this is not why they have limited use in targeting negative symptoms. The actual reason is that antipsychotic medications have very limited efficacy in reducing negative symptom severity and lack established evidence for treating primary and enduring negative symptoms. This is precisely the problem facing therapeutic research for negative symptoms, that is, the lack of evidence for available interventions. Kindly refer to:
o Fusar-Poli P, Papanastasiou E, Stahl D, et al. Treatments of Negative Symptoms in Schizophrenia: Meta-Analysis of 168 Randomized Placebo-Controlled Trials [published correction appears in Schizophr Bull. 2022 May 7;48(3):721. doi: 10.1093/schbul/sbz071.]. Schizophr Bull. 2015;41(4):892-899. doi:10.1093/schbul/sbu170
o Krause M, Zhu Y, Huhn M, et al. Antipsychotic drugs for patients with schizophrenia and predominant or prominent negative symptoms: a systematic review and meta-analysis. Eur Arch Psychiatry Clin Neurosci. 2018;268(7):625-639. doi:10.1007/s00406-018-0869-3
o Remington G, Foussias G, Fervaha G, et al. Treating Negative Symptoms in Schizophrenia: an Update. Curr Treat Options Psychiatry. 2016;3:133-150. doi:10.1007/s40501-016-0075-8

**Reply4:**

Thank you for your insightful comments, the provided references, and for highlighting the need to clarify the limitations of antipsychotic medications in targeting negative symptoms. We agree that the limited efficacy of antipsychotics in reducing the severity of negative symptoms, particularly primary and enduring ones, is a critical issue. This indeed represents a significant challenge in schizophrenia treatment research. In response to your feedback, we have revised the introduction and incorporated citations to the relevant literature you provided to better reflect this point. We believe these changes provide a more accurate and comprehensive discussion of the challenges in treating negative symptoms and the limitations of current interventions. Once again, we sincerely appreciate your valuable input, which has significantly improved the quality of our manuscript.

**Changes in the text:** However, current evidence indicates that the efficacy of antipsychotic medications in reducing the severity of negative symptoms is highly limited, and there is a lack of robust evidence for their effectiveness in treating primary and enduring negative symptoms. This represents a core challenge in current research on the treatment of negative symptoms, while also highlighting the insufficient evidence supporting existing interventions. **(Introduction: see marked manuscript Page 5, line 108-113)**

**Comment 5:**

In the results, the authors state that “The subgroup differences (P = 0.68).” I am not sure what this means. The subgroup analysis indicates that the reduction in negative symptoms is not consistently significant across time. In the abstract and discussion, the authors argue that this lack of effect across time is “due to the lack of cognitive therapy specifically targeting negative symptoms.” This is merely a conjecture. The results, in my view, suggest that CBT and CRT do not effectively improve primary and enduring negative symptoms, which are the main targets of negative symptom treatment in schizophrenia. This needs to be mentioned as a possible interpretation of the results. After all, secondary negative symptoms in schizophrenia also respond to antipsychotic medications. Please see:
o Correll CU, Schooler NR. Negative Symptoms in Schizophrenia: A Review and Clinical Guide for Recognition, Assessment, and Treatment. Neuropsychiatr Dis Treat. 2020;16:519-534. Published 2020 Feb 21. doi:10.2147/NDT.S225643

**Reply5:**

Thank you for your valuable comments and suggestions. We truly appreciate your careful review of our manuscript and have taken the issues you raised into consideration.

We have revised the results section to reflect that Cognitive Behavioral Therapy (CBT) significantly improved negative symptoms of schizophrenia in the short, medium, and long term (all P<0.05). We are grateful for your emphasis on the importance of distinguishing between primary and secondary negative symptoms. As you suggested, we have incorporated a perspective that our results may indicate limited efficacy of CBT in treating primary, enduring negative symptoms (such as intrinsic deficits in motivation or emotional expression). We have elaborated on the potential implications of our findings for both primary and secondary negative symptoms in the discussion section. We will also draw on the research by Correll and Schooler (2020) to provide a more in-depth analysis.

Thank you once again for your insightful feedback. We believe these revisions have strengthened our manuscript.

**Changes in the text: (Discussion: see marked manuscript Page 14-16 line 399-494)**

**Comment 6:**

Also related to the previous point is the finding in this systematic review that total PANSS scores tend to decrease with CBT or CRT, which further raises the possibility that improvement in negative symptoms is linked to improvement in other domains—that is, the effect may primarily be on secondary negative symptoms. This issue needs to be discussed in detail. Unfortunately, due to this problem, the results do not advance the field of negative symptom therapeutics, except for the finding that there is no significant difference across short- and long-term comparisons, which, in my view, indicates a lack of benefit.

**Reply6:**

Thank you for your comments, and we appreciate your raising the important issue of the potential link between the improvement of negative symptoms and other domains (such as secondary negative symptoms). We are grateful for your careful consideration of this issue and have revised our manuscript to address it in detail. Upon re-analyzing the data, we found that Cognitive Behavioral Therapy (CBT) can indeed lead to consistent and significant improvements in negative symptoms of schizophrenia across short-term, medium-term, and long-term follow-ups. The observed improvements in negative symptoms are sustained and significant over time, supporting the potential efficacy of CBT in addressing both primary and secondary negative symptoms.We have added a detailed discussion of this issue in the revised manuscript, including the need for further research to distinguish the effects on primary versus secondary negative symptoms. We believe that these revisions provide a more nuanced and evidence-based discussion of our results.

Once again, we sincerely thank you for your constructive feedback, which has greatly enhanced the quality of our manuscript.

**Changes in the text: (Results:** **see marked manuscript Page 9-10 line 273-288. Discussion: see marked manuscript Page 14-16 line 399-494. Conclusions: see marked manuscript Page 17 line 528-536.)**

We tried our best to improve the manuscript, and made some changes in the manuscript. These changes will not influence the content and framework of the paper. We appreciate reviewers' and editors' warm work earnestly, and hope that the correction will meet with the approval. Once again, thank you very much for your comments and suggestions. Please don’t hesitate to contact us in case there are any problems regarding this manuscript.

Best wishes,

Yu Hong

Guangzhou Huali College
