# Supplementary material for: Cognitive-behavioral therapy for the improvement of negative symptoms and functioning in schizophrenia: A systematic review and meta-analysis of randomized controlled trials
Source: PLoS One. 2025 May 20;20(5):e0324685. doi: 10.1371/journal.pone.0324685 (PMC12091889; doi:10.1371/journal.pone.0324685)
Supplement: Attachment — Submitted filename: Response_to_Reviews_auresp_2.docx [file pone.0324685.s012.docx]

Dear Editors and Reviewers:

Thank you for your letter and for the reviewers' comments concerning our manuscript entitled “Cognitive-behavioral therapy for the improvement of negative symptoms and functioning in schizophrenia: a systematic review and meta-analysis of randomized controlled trials” (Manuscript ID: PONE-D-24-37272R1). Those comments are all valuable and very helpful for revising and improving our paper, as well as the important guiding significance to our research. We have studied comments carefully and have made correction which we hope to meet with approval. Revised portion are marked in red (with track and highlighted changes) in the paper. The main corrections in the paper and the responses to the reviewer’s comments are as follows. Please don’t hesitate to contact us in case there are any problems regarding this manuscript.

**Reviewer #1**

**Comment 1:**

Thank you for the opportunity to review this interesting paper and taking into account the previous comments and making changes accordingly.

**Reply 1:**

Thank you very much for your positive feedback and for acknowledging the changes we have made. Your constructive comments have been invaluable in enhancing the quality of our manuscript. We truly appreciate your time and effort in reviewing our work.

**Reviewer #2**

**To begin with, we thank the reviewer for the effort and time put into the review of the manuscript, special thanks to you for your good comments and warm work earnestly.**

**General comments:**

 I would like to thank the authors for their major revision of the manuscript and for considering my suggestion to restrict their systematic review and meta-analysis to CBT and focus on negative symptoms. This has significantly strengthened the manuscript and the conclusions drawn from their analysis. The authors have addressed all the issues raised in the previous round of review and now provide a more robust discussion that situates their results within the literature on negative symptom treatment in schizophrenia. I have a few comments I believe the authors should consider:

**Reply:**

Thank you very much for your positive and constructive feedback on our revised manuscript. We are pleased that you found the changes we made to be significant improvements, particularly in focusing the systematic review and meta-analysis on CBT and negative symptoms. Your suggestion has indeed strengthened the manuscript and helped us draw more robust conclusions.

We have carefully considered your additional comments and have addressed them in the latest revision of our manuscript. We believe these changes further enhance the clarity and relevance of our discussion within the context of negative symptom treatment in schizophrenia.

Thank you once again for your valuable insights and for taking the time to review our work. We truly appreciate your support and guidance.

**Comment 1:**

The authors continue to refer to “cognitive abilities” as constituting negative symptoms. This is incorrect, as I explained in detail in my previous review. I suggest this be revised in both the abstract and the introduction.

**Reply1:**

Thank you for your continued attention to detail and for bringing this important point to our attention once again. We have carefully reviewed the sections of our manuscript where we previously referred to “cognitive abilities” as constituting negative symptoms and have made the necessary revisions to correct this inaccuracy.

We have updated both the abstract and the introduction to accurately reflect the distinction between cognitive abilities and negative symptoms, ensuring that our terminology is precise and consistent with the established definitions in the literature. We appreciate your guidance on this matter and believe that these changes have further improved the accuracy and clarity of our manuscript.

Thank you once again for your valuable feedback.

**Changes in the text:** Negative symptoms of schizophrenia are a range of deficits or losses in mental functioning associated with the disorder, including blunted affect, alogia, avolition, asociality, and anhedonia. **(Abstract: see marked manuscript Page 1-2 line 23-35)**

**Comment 2:**

In the introduction, the authors state that “there is a notable absence of systematic reviews and meta-analyses that specifically address the improvement of negative symptoms.” This is also incorrect. As I pointed out in my earlier review, there is a recent systematic review and meta-analysis that addressed this same question:
Xu, Feifei, and Sheng Xu. “Cognitive-behavioral therapy for negative symptoms of schizophrenia: A systematic review and meta-analysis.” Medicine 103, no. 36 (2024): e39572. doi:10.1097/MD.0000000000039572.
I suggest the authors explicitly justify the contribution of their study in light of this prior work. One possible approach is to highlight the aspects uniquely addressed in the current review, such as the differentiation of short-, medium-, and long-term effects, as well as the inclusion of functioning as an outcome.

**Reply2:**

Thank you very much for your detailed and constructive feedback. We appreciate your patience and the effort you have put into helping us improve our manuscript.

We have carefully reviewed the introduction and have revised the statement to accurately reflect the existing literature. We now explicitly acknowledge the recent systematic review and meta-analysis by Xu and Xu (2024) and have provided a clear justification for the unique contributions of our study.

In our revised introduction, we have highlighted the specific aspects that differentiate our review from the prior work, such as the detailed differentiation of short-, medium-, and long-term effects, as well as the inclusion of functioning as an outcome. We believe these aspects provide valuable additional insights and contribute to the broader understanding of cognitive-behavioral therapy for negative symptoms in schizophrenia.

We have also added a citation to the Xu and Xu (2024) study to ensure proper acknowledgment of their work. We hope these revisions address your concerns and provide a clearer context for our study.

Thank you once again for bringing this important point to our attention and for providing the reference. Your feedback has been invaluable in enhancing the accuracy and relevance of our manuscript.

**Changes in the text: (Introduction: see marked manuscript Page 6, line 148-159)**

**Comment 3:**

The authors report a mean difference in the abstract, but should clarify that this pertains to the PANSS Negative Symptom subscale.
**Reply3:**

Thank you for your careful review and for pointing out the need for clarification in the abstract. We have revised the abstract to explicitly state that the reported mean difference pertains to the PANSS Negative Symptom subscale. This change ensures that our findings are clearly understood and accurately represented.

We appreciate your attention to detail and your suggestions for improving the clarity of our manuscript.

**Changes in the text:** The analysis included a total of 15 studies involving 1,311 participants. All studies used the Positive and Negative Syndrome Scale (PANSS) as the assessment tool for measuring negative symptoms of schizophrenia. **(Abstract: see marked manuscript Page 4, line 68-70)**

We sincerely appreciate the efforts of both the editors and reviewers for their valuable feedback and guidance throughout the process. Your expertise has been instrumental in improving our manuscript.

Best wishes,

Yu Hong

Guangzhou Huali College
